# The role of the ecological scaffold in the origin and maintenance of whole-group trait altruism in microbial populations

**DOI:** 10.1186/s12862-023-02112-2

**Published:** 2023-04-12

**Authors:** C. T. Jones, L. Meynell, C. Neto, E. Susko, J. P. Bielawski

**Affiliations:** 1 Department of Biochemistry and Molecular Biology, Dalhousie University, NS Halifax, Canada; 2 Department of Philosophy, Dalhousie University, Halifax, Canada; 3 Department of Social and Political Sciences, Philosophy, and Anthropology, University of Exeter, Exeter, UK; 4 Centre for the Study of the Life Sciences, EGENIS, University of Exeter, Exeter, UK; 5 Department of Mathematics and Statistics, Dalhousie University, Halifax, Canada; 6 Department of Biology and Dept. of Mathematics and Statistics, Dalhousie University, Halifax, Canada

**Keywords:** Ecological scaffold, Whole-group trait altruism, Kin selection, Multilevel selection, Population structured selection, Strong and weak altruism, Random and positive assortment, Nutrient limitation

## Abstract

**Background:**

Kin and multilevel selection provide explanations for the existence of altruism based on traits or processes that enhance the inclusive fitness of an altruist individual. Kin selection is often based on individual-level traits, such as the ability to recognize other altruists, whereas multilevel selection requires a metapopulation structure and dispersal process. These theories are unified by the general principle that altruism can be fixed by positive selection provided the benefit of altruism is preferentially conferred to other altruists. Here we take a different explanatory approach based on the recently proposed concept of an “ecological scaffold”. We demonstrate that ecological conditions consisting of a patchy nutrient supply that generates a metapopulation structure, episodic mixing of groups, and severe nutrient limitation, can support or “scaffold” the evolution of altruism in a population of microbes by amplifying drift. This contrasts with recent papers in which the ecological scaffold was shown to support selective processes and demonstrates the power of scaffolding even in the absence of selection.

**Results:**

Using computer simulations motivated by a simple theoretical model, we show that, although an altruistic mutant can be fixed within a single population of non-altruists by drift when nutrients are severely limited, the resulting altruistic population remains vulnerable to non-altruistic mutants. We then show how the imposition of the “ecological scaffold” onto a population of non-altruists alters the balance between selection and drift in a way that supports the fixation and subsequent persistence of altruism despite the possibility of invasion by non-altruists.

**Conclusions:**

The fixation of an altruistic mutant by drift is possible when supported by ecological conditions that impose a metapopulation structure, episodic mixing of groups, and severe nutrient limitation. This is significant because it offers an alternative explanation for the evolution of altruism based on drift rather than selection. Given the ubiquity of low-nutrient “oligotrophic” environments in which microbes exist (e.g., the open ocean, deep subsurface soils, or under the polar ice caps) our results suggest that altruistic and cooperative behaviors may be highly prevalent among microbial populations.

**Supplementary Information:**

The online version contains supplementary material available at 10.1186/s12862-023-02112-2.

## Background

There has been a long-standing debate between kin selection and multilevel selection as explanations for the evolution of altruism. The current consensus appears to be that the two theories amount to the same thing, differing mostly in their mathematical details [1–6]. Both approaches appeal to some form of “population structured selection” [7], the salient point being that, whereas the fixation of an altruistic mutant is unlikely in an unstructured population, it is much more likely when population structure causes the benefit of altruism to be preferentially conferred to other altruists. Recent publications define an “ecological scaffold” to be a set of environmental factors that increase the probability of an evolutionary outcome that would otherwise be unlikely (cf. [8–10]). The notion was initially developed to provide an explanation for the evolution of multicellular forms starting from populations of single cells. Yet it is quite useful in explanations for the evolution of traits such as altruism. Here we show that nutrient limitation, when combined with population structured selection based on spatial structure and patterns of dispersal, can play a crucial role in the origin and maintenance of a type of altruism commonly observed among microbes.

### Kin selection and Hamilton’s rule

In evolutionary theory, altruism refers to a behavior that reduces the fitness of the individual exhibiting that behavior but increases the fitness of others in the same group or population (see [11] for a compendium of definitions related to altruism and cooperation). A classical example is sentinel behavior, where some individuals stand watch over a kin group and issue a warning call when a predator is detected. Such behavior exposes the sentinel to an increased risk of death by predation but also reduces that risk to others in the group. How can such a behavior, so costly to the altruistic individual, evolve?

It has recently been demonstrated that meerkats in captivity exhibit sentinel behavior despite a lack of predators in their environment [12]. This underscores the fact that altruistic behavior is often innate and determined by genes to some degree. The gene-centric explanation goes as follows (cf. [13, 14]). The sentinel behavior comes at a cost in the form of a reduction in the probability that the gene or gene complex ($G$) responsible for that behavior will be replicated when its host organism produces offspring. But there is also a benefit in the increased probability that $G$ will be replicated when other individuals in the kin group reproduce, some proportion of which carry $G$. Hence, the change in the relative frequency of $G$ over one generation depends on a combination of the direct effect (the cost $C$), and the indirect effect (the benefit $B$) of the altruistic behavior scaled by a coefficient of relatedness ($r$).

The impact of cost, benefit, and relatedness^1^ on selection is encapsulated by Hamilton’s rule, which states that a gene for altruism can proliferate in a population if the scaled benefit outweighs the cost, $rB-C>0$ [19]. Individual traits that increase the value of $r$ play a central role in kin selection theory. Prominent among these is kin recognition, where altruists can recognize other altruists, and limited dispersal, where altruistic individuals tend to remain in proximity to one another. The inclusive fitness $rB-C$ of an altruist (or, for gene-centrists, of $G$), is increased by these effects to the extent that they cause altruists to preferentially confer their benefit to other altruists. See Frank [20] for a history of kin selection theory.

A common scenario of altruism among microbes occurs when an individual produces a public good that increases the fitness of others in the same population. An archetypical example is the production of iron-scavenging siderophores, which make chelated iron available as a public good but are costly to produce [21, 22]. The gene or gene complex $G$ responsible for this behavior might persist despite the cost in a viscous population in which dispersal is limited^2^ [19] or in a spatially unstructured population if $G$ also confers the ability to alter behavior in response to environmental cues in a way that preferentially confers the benefit of altruism to other altruists (i.e., by some form of quorum sensing [26, 27]).

### Population structured selection

The spatial distribution of genetic variants is largely determined by the way individuals move or are moved from one location to another (i.e., patterns of dispersal). This is often a function of life history or traits that are endogenous to individuals. The production of sticky substances, for example, can limit dispersal and lead to the formation of microbial mats as one part of a life cycle (e.g., [28]). Structure can also be exogenously imposed by features of the environment. A typical scenario arises when the supply of essential resources is patchy and a population is separated into distinct groups (i.e., a metapopulation). It is often assumed that such groups episodically exchange individuals in a way that reflects differences in group size. Scenarios of this kind are ubiquitous in discussions of multilevel selection [29–34] and studies related to Wright’s shifting balance theory [35, 36].

Population structured selection (cf. [7, 37]) refers to any scenario in which spatial structure, patterns of dispersal, and/or patterns of interaction between individuals cause the frequency distribution of genotypes to change over time in a way that is different from what would be expected based on difference in fitness alone (e.g., [38]). Here we focus our attention on scenarios in which a population is spatially structured with some form of dispersal. A prototypical scenario under kin selection theory is a viscous population in which selection is based on the amplification of the coefficient of relatedness ($r$). The standard scenario under multilevel selection is trait-group selection [29], where all individuals from ancestral groups are episodically collected into a common pool from which new descendant groups are randomly drawn. In this case selection is based on a positive correlation between the mean fitness of all individuals within each group and the proportion of altruists they contain [33].

### Weak and strong altruism

The terms “strong” and “weak” altruism were introduced by Wilson [39]. More recently, strong altruism has been defined as the scenario under which a non-altruistic individual, if it were to convert to an altruist, would suffer a loss in fitness; altruism is otherwise weak^3^ [33, 41]. Consider whole-group trait altruism in which an individual produces a public good that benefits all members of its population, including itself [22]. Such an altruist suffers a cost but also accrues a direct benefit from its action. Whole-group trait altruism can therefore be strong or weak depending on the balance between cost and benefit. This is usually contrasted with other-only trait altruism [22], which benefits others but not the altruist itself and can therefore only be strong.

Whether strong altruism is favored in a spatially structured population often depends on the nature of the dispersal process. A metapopulation structure provides a means by which variation in the proportion of altruists between groups (${\pi }_{k}$ for the ${k}^{th}$ group) can arise by a combination of mutation, selection, and drift. Dispersal then provides the opportunity for such variation to affect a change in the proportion of altruists in the metapopulation ($\pi$). Altruism is more strongly favored when the variance in the proportion of altruists between groups is larger ($\mathrm{Var}\left({\pi }_{k}\right)>0$) and the expected variance within groups is negligible (i.e., $\mathrm{E}\left({\pi }_{k}\left(1-{\pi }_{k}\right)\right)\approx 0$) ([33], p. 194). Any process that gives rise to this population structure, where each individual group tends to be homogeneous but there is heterogeneity between groups, can increase the likelihood that strong altruism will be fixed in the metapopulation.

Consider again trait-group selection, where individuals are episodically pooled and then randomly assorted into new groups. The expected proportion of altruists within each newly formed group is equal to the proportion of altruists in the common pool. Pooling-and-redistribution will therefore reduce $\mathrm{Var}({\pi }_{k})$ toward zero apart from small differences due to the stochasticity of the redistribution process. For this reason, trait-group selection cannot support the evolution of strong altruism, although it can support weak altruism ([15, 33], p. 192–197, [40]). Strong altruism can be supported by a dispersal process that positively assorts altruists, causing altruists to be grouped together with a probability greater than chance [3, 42]. Such a process can increase $\mathrm{Var}({\pi }_{k})$, reduce $\mathrm{E}\left({\pi }_{k}\left(1-{\pi }_{k}\right)\right)$, and thereby support the fixation of altruism in the metapopulation.^4^

### The origin and maintenance of whole-group trait altruism

The preceding suggests that a single group of strong altruists can displace groups of non-altruists in a metapopulation provided the dispersal process positively assorts altruistic individuals. Suppose, however, that each group in the metapopulation was spatially unstructured. That would make it difficult to explain how that one group of strong altruists might arise in the first place. This is where the notion of an “ecological scaffold” is relevant [8–10, 45]. The models that emerged from the modern synthesis often assume a single isolated and spatially unstructured population with random mating in which altruism cannot evolve by selection (e.g., a classic Wright-Fisher population, [35, 38, 46, 47]). The environment is taken for granted or assigned to the background of such models. An ecological scaffold, by contrast, puts the environment into the foreground by accounting for ecological features that can change the probability of evolutionary outcomes. The scaffolding concept is arguably implicit in existing explanations for the evolution of altruism, at least insofar as spatial structure and dispersal processes are assumed to be imposed by environmental conditions. Yet traditional models often overlook the potential role of interactions between spatial structure and dispersal and other aspects of the environment such as nutrient limitation.^5^

Here we illustrate how nutrient limitation can play a key role as one component of an ecological scaffold for the origin and maintenance of whole-group trait altruism. To this end, we imagine an environment that imposes a metapopulation structure and dispersal process with random assortment onto a population of non-altruists. We show that a whole-group trait altruist that arises in any one group by mutation will almost certainly be eliminated when nutrients required for growth are plentiful, and that even if altruism was fixed in one group it would not survive for long because the nutrient regime makes whole-group trait altruism strong. The situation changes when the supply of nutrients is limited to such an extent that the fitness advantage enjoyed by the non-altruist is decreased due to the softening of selection, and the magnitude of drift is increased due to the reduction in group size. These ecological effects make it possible for a whole-group trait altruist mutant to be fixed within any one group by drift alone. We go on to show that nutrient limitation not only makes whole-group trait altruism effectively weak but can also generate a population structure in which $\mathrm{Var}\left({\pi }_{k}\right)>0$ and $\mathrm{E}\left({\pi }_{k}\left(1-{\pi }_{k}\right)\right)\approx 0$. These conditions make it possible for a single group of altruists to propagate via a randomly assorting dispersal process and become fixed in the metapopulation by drift alone.

## Results

We assume a microbial population composed of an S-type (non-altruistic or selfish)^6^ that is characterized by the rate ${c}_{S}$ at which it consumes a growth-limiting nutrient $R$ (resource) per cell per generation. An A-type (altruist) is an S-type mutant that suffers a lower consumption rate ${c}_{A}<{c}_{S}$ but improves the local environment by producing a public good [21, 22] or by regulating an abiotic state such as local temperature or pH [53], such effects being determined by the presence or activation of gene complex $G$. The public good is assumed to benefit both types by reducing the common death rate to a degree that increases with the proportion of A-types in the population ($\pi$). A cell of either type will reproduce when it consumes one unit of $R$. The S-type therefore reproduces faster and is the fitter of the two because of its higher consumption rate. The deficit in consumption rate $\delta ={c}_{A}{/c}_{S}<1$ accounts for the cost of the altruistic behavior.

### Stochastic simulations in a single population

Our first objective was to estimate the probability that a single A-type mutant in an S-type population might be fixed by drift. Each simulation started with an S-type population into which one S-type was converted to an A-type. The stochastic population model was then run until the A-type was either fixed or eliminated. This was repeated ${10}^{5}$ times. Simulations were conducted under a relatively high nutrient influx ${R}_{in}=50$ units per generation, and under “severe” nutrient limitation with ${R}_{in}=5$ units per generation. The fitness deficit suffered by A-type cells was set to $\delta =0.98$ or $\delta =1.00$, the latter being used to assess the probability of fixation under neutrality. A similar set of simulations was conducted starting with an A-type population in which one A-type was converted to an S-type. Results are summarized in Table 1.

**Table 1:** Estimated probabilities of fixation within a single population

	Estimated probability that an A-type mutant is fixed		Estimated probability that an S-type mutant is fixed	
	$\delta =0.98$	$\delta =1.00$	$\delta =0.98$	$\delta =1.00$
${R}_{in}=50$	${4.0\times 10}^{-5}$	${6.0\times 10}^{-3}$	${1.6\times 10}^{-2}$	${1.1\times 10}^{-3}$
${R}_{in}=5$	${4.4\times 10}^{-2}$	${5.8\times 10}^{-2}$	${1.6\times 10}^{-2}$	${1.0\times 10}^{-2}$

An A-type mutant in an S-type population was unlikely to be fixed when ${R}_{in}=50$ and $\delta =0.98$ (estimated probability ${4.0\times 10}^{-5}$) and its probability of fixation was two orders of magnitude less than the probability under neutrality (${6.0\times 10}^{-3}$). When ${R}_{in}=5$ the probability of A-type fixation was not only much larger (${4.4\times 10}^{-2}$) but also just slightly less than the probability under neutrality (${5.8\times 10}^{-2}$). The probability that an S-type mutant in an A-type population was fixed was ten times greater than the probability under neutrality when ${R}_{in}=50$ (${1.6\times 10}^{-2}$ vs ${1.1\times 10}^{-3}$). But the two probabilities were nearly the same when ${R}_{in}=5$ (${1.6\times 10}^{-2}$ vs ${1.0\times 10}^{-2}$). These results illustrate how changes in the balance between selection and drift caused by severe nutrient limitation can give rise to a nearly neutral selection regime in which an A-type mutant is reasonably likely to be fixed despite the cost incurred by producing the public good.

#### Nutrient limitation and macroevolutionary dynamics

The results in Table 1 provide estimates for the probability that a population will undergo a transition from one type (e.g., selfish) to the other type (e.g., altruistic) by a combination of mutation, selection, and drift. Let us now suppose that cells can mutate from one type to the other with some small probability (e.g., ${P}_{mut}={10}^{-6}$ per cell per generation) via a simple “genetic switch” (cf. [28, 54]). This assumption gives rise to an interesting dynamic over macroevolutionary time. Let ${P}_{AS}$ represent the probability that an A-type population will transition to an S-type population by selection following an A-to-S-type mutation in a single A-type cell. Similarly, let ${P}_{SA}$ represent the probability that an S-type population will transition to an A-type population by drift following an S-to-A-type mutation in a single S-type cell. Each of these probabilities is just the product of the probability of mutation per cell (${P}_{mut}$), the number of cells in the population (${N}_{cap}$), and the probability that the mutant reaches fixation (${P}_{S}^{fix}$ and ${P}_{A}^{fix}$):

$${P}_{AS}={P}_{mut}{N}_{cap}(\pi =1) {P}_{S}^{fix}, {P}_{SA}={P}_{mut}{{N}_{cap}(\pi =0) P}_{A}^{fix}$$

Here ${N}_{cap}(\pi =1)$ and ${N}_{cap}(\pi =0)$ represent the size an A-type and S-type population at birth–death equilibrium (i.e., at carrying capacity or “cap”). Using the values in Table 1 (those for $\delta =0.98$), an A-to-S transition is more than two thousand times more likely than an S-to-A transition when ${R}_{in}=50$ (${P}_{AS}/{P}_{SA}\approx 2400$) but only about twice as likely when ${R}_{in}=5$ (${P}_{AS}/{P}_{SA}\approx 2$). This result underlines the role severe nutrient limitation can play in explaining the origin of an A-type population. But it also emphasizes how vulnerable an A-type population is to S-type mutants even when ${R}_{in}=5$ (since ${P}_{AS}>{P}_{SA}$).

**Fig. 1:**
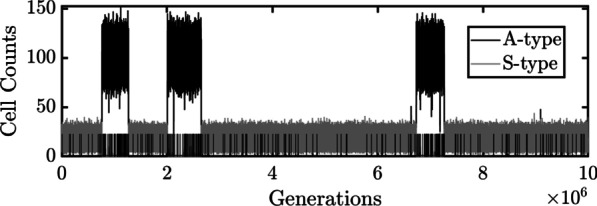
Macroevolutionary Dynamics when ${R}_{in}=5$. Each cell is assumed to have a small probability of mutating to a cell of the opposite type. Under this condition, and when ${R}_{in}=5$, a single unstructured population or group will episodically transition between an A-type population with $\pi =1$ and an S-type population with $\pi =0$. Mutants of both types arise randomly (as indicated by vertical lines) and are usually eliminated. When fixation does occur, it does so very quickly due to the small size of the population, ${N}_{cap}\left(\pi =0\right)=16$ S-type cells and ${N}_{cap}\left(\pi =1\right)=100$ A-type cells. The population is therefore bistable but spends more time in the S-type state due to the fitness deficit suffered by A-type cells

A single run of the stochastic population model conducted with ${R}_{in}=5$ is shown in Fig. 1. The state of the population (selfish or altruistic) is indicated by the mean cell count, ${N}_{cap}\left(\pi =0\right)=16$ cells for an S-type population and ${N}_{cap}\left(\pi =1\right)=100$ cells for an A-type population (see Methods). Variations about these means reflect the stochasticity of the birth and death processes. The difference in population size explains why mutations, indicated by vertical lines along the horizontal axis, arose more frequently when the population was altruistic. Notice that mutants were fixed or eliminated very rapidly. This is significant because it means that the two types seldom coexisted (cf. [51]). The fact that the population transitioned several times to the altruistic state shows how whole-group trait altruism can arise in a single unstructured population by mutation and drift. However, each time the population transitioned to the altruistic state, it was not long before it transitioned back.

#### Dispersal by migration

The fixation of a slightly inferior variant by drift is a phenomenon that is well understood. The importance of the single population simulation above is that it shows that, although an A-type population can arise under severe nutrient limitation, it is unlikely to persist in the face of S-type mutants. Persistence requires either a change in the intrinsic properties of A-type cells (e.g., the evolution of some form of quorum sensing) or the imposition of supportive environmental conditions. Our second objective was to explore the impact of spatial segregation and dispersal on the evolution of whole-group trait altruism in accordance with scenarios typically considered under multilevel selection theory.

The dispersal process cited most under multilevel selection theory is trait-group selection, whereby all individuals in a metapopulation are episodically pooled and redistributed. This model was intended to mimic the life-history of species in which “individuals are spatially restricted during most of their life cycle, with the exception of their dispersal phase” [29]. An alternative form of dispersal is migration, wherein individuals drawn from a donor group are transferred into a recipient group (e.g., [36, 55]). Here we consider the scenario in which individuals are drawn from a donor group, either chosen randomly or by a selective criterion, in proportion ($p$) to the size of that group. The resulting “migration propagule” is then transferred into a recipient group selected at random. Migrants from the donor group and residents within the recipient group are therefore mixed in the location of the recipient group (i.e., in the “recipient patch”).

The expected number of generations before the fixation of a nearly neutral mutation is on the order of the effective size of the population [56]. The fixation or elimination of a mutant when ${R}_{in}=5$ is therefore very rapid. Hence, groups in a metapopulation will tend to be composed of a single genotype, making $\mathrm{E}\left({\pi }_{k}\left(1-{\pi }_{k}\right) \right)\approx 0$. At the same time, the possibility of A-type fixation within groups by drift makes $\mathrm{Var}\left({\pi }_{k}\right)>0$, with ${\pi }_{k}=1$ in some groups and ${\pi }_{k}=0$ in others. This population structure, when combined with migration, has a significant impact on the evolution of the metapopulation. If migrants and residents within a recipient group are of opposite types, then one type will be fixed in a few tens of generations (cf. Fig. 1). The probability that the A-type is fixed by drift in a recipient group is an increasing function of the proportion of A-types the recipient group contains. This probability is maximized when one group is A-type (${\pi }_{k}=1$) and the other is S-type (${\pi }_{k}=0$) because A-type groups (${N}_{cap}=100$ cells) are so much larger than S-type groups (${N}_{cap}=16$ cells).

**Fig. 2:**
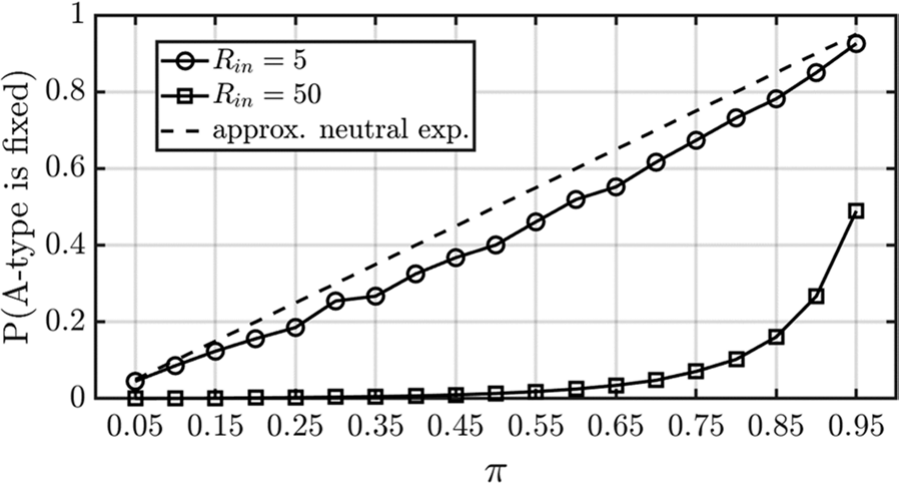
The probability that the A-type will be fixed in a new recipient group as a function of $\pi$. Estimates for the probability of A-type fixation were generated by running the stochastic population model on populations with $\pi \in \left\{0.05, 0.10,\dots , 0.95\right\}$ for each value of ${R}_{in}\in \left\{5, 50\right\}$. Each $\left(\pi ,{R}_{in}\right)$ pair was run ${10}^{5}$ times. Probabilities under neutrality (i.e., when $\delta =1$) are approximated by the dashed one-to-one line. Note that $\pi$ is the proportion of A-types in a recipient group regardless of the identity of that group. It therefore plays the role of either ${\pi }_{AS}$ or ${\pi }_{SA}$ when predicting the probability of A-type fixation

Estimates for the probability that the A-type is fixed in a mixed group are shown in Fig. 2. The probability of fixation is quite low for all values of $\pi <0.85$ when ${R}_{in}=50$. But when the nutrient influx is reduced to ${R}_{in}=5$, the probability that the A-type is fixed is very close to the probability under neutrality, as approximated^7^ by $\pi$ and indicated by the dashed one-to-one line. To take an example, suppose the size of a migration propagule is $p=0.25$ times the size of the donor group. Let ${\pi }_{AS}$ represent the expected proportion of A-type cells in a recipient S-type group after it has received a migration propagule from an A-type donor. And let ${\pi }_{SA}$ represent the expected proportion of A-type cells in a recipient A-type group after it has received a migration propagule from an S-type donor. These proportions work out to ${\pi }_{AS}=0.60$ and ${\pi }_{SA}=0.96$ assuming model parameters that make an A-type group six times larger than an S-type group (see Methods). The corresponding estimates of the probabilities of fixation obtained using the data in Fig. 2 are shown in Table 2.

**Table 2:** Estimated probabilities of fixation following migration

$p=0.25$	Estimated probability that an A-type donor group is re-produced	Estimated Probability that an S-type donor group is re-produced
${R}_{in}=50$	$0.03$	$0.50$
${R}_{in}=5$	$0.52$	$0.07$

A donor group is reproduced, in a manner of speaking (let us say “re-produced”^8^), when its migration propagule is fixed in a recipient group of the opposite type. Reading from Table 2, an A-type donor is re-produced in an S-type recipient patch with probability $0.03$ when $p=0.25$ and ${R}_{in}=50$. Under the same conditions, an S-type donor is re-produced in an A-type recipient patch with probability $0.50$. The migration process therefore amplifies the fitness advantage enjoyed by S-type cells when the nutrient influx is relatively high. When ${R}_{in}=5$, by contrast, an A-type donor is re-produced with probability $0.52$ compared to only $0.07$ for an S-type donor. In this case the migration process favors the A-type. This is partly due to the impact severe nutrient limitation has on the balance between selection (which is weakened) and drift (which is amplified), but also to the fact that A-type groups are so much larger than S-type groups. A single A-type mutant that arises in an S-type group is fixed with probability $4.4\times {10}^{-2}$ when ${R}_{in}=5$ (Table 1). But an A-type migration propagule is fixed in an S-type recipient patch with probability $0.52$. The “weight of numbers” advantage enjoyed by A-type groups therefore plays an important role in overcoming the cost of whole-group trait altruism.

### Random and selective migration in a metapopulation

Let ${F}_{A}$ represent the fraction of A-type groups in a metapopulation with a constant number of groups (here assuming groups with either $\pi =0$ or $\pi =1)$. The nominal fitness ($W$) of a group^9^ is the product of the probability that it is selected to be a donor, the probability that the recipient group is of the opposite type, and the probability that its migration propagule is fixed in the recipient group. For example, when ${R}_{in}=5$ and the donor and recipient groups are randomly selected (the random migration model):

$${W}_{A}\cong 0.52{F}_{A}\left(1-{F}_{A}\right), {W}_{S}\cong 0.07{F}_{A}\left(1-{F}_{A}\right)$$

The difference between these values is positive, ${W}_{A}-{W}_{S}\cong 0.45{F}_{A}\left(1-{F}_{A}\right)$. It follows that the A-type, starting from a single A-type group, can propagate across the metapopulation and displace the S-type under the random migration process. When ${R}_{in}=50$ the difference is negative, ${W}_{A}-{W}_{S}\cong -0.47{F}_{A}\left(1-{F}_{A}\right)$. In this case, the A-type will almost certainly be eliminated. Suppose, however, that the migration process was constrained so that only A-type groups can be donors (the selective migration model, cf. [36]). This makes ${W}_{S}=0$ and ${W}_{A}-{W}_{S}=0.03{F}_{A}\left(1-{F}_{A}\right)$. Selective migration therefore makes it possible for a single A-type group to propagate across an S-type metapopulation even when ${R}_{in}=50$. Using these results, we can predict that the A-type has some chance of being fixed in the metapopulation under random migration when ${R}_{in}=5$ but that fixation requires selective migration when ${R}_{in}=50$.

#### Stochastic simulations in a metapopulation

Simulations were conducted to test our theoretical predictions about the fate of the A-type in a metapopulation setting. The first (Sim 1) was designed to test whether a single A-type group in an otherwise S-type metapopulation might reach fixation under Selective Migration (SM) but not under Random Migration (RM) or Trait-group Selection (TG) when nutrients are relatively abundant (${R}_{in}=50$). The objective of the second (Sim 2) was to test the prediction that an S-type metapopulation will tend toward a mix of A-type and S-type groups in dynamic equilibrium in the absence of dispersal when nutrients are severely limited (${R}_{in}=5$). The third (Sim 3) was then conducted to test whether a single A-type mutant might reach fixation in the metapopulation under RM or TG when nutrients are severely limited (${R}_{in}=5$), when selection within groups is nearly neutral.

**Fig. 3:**
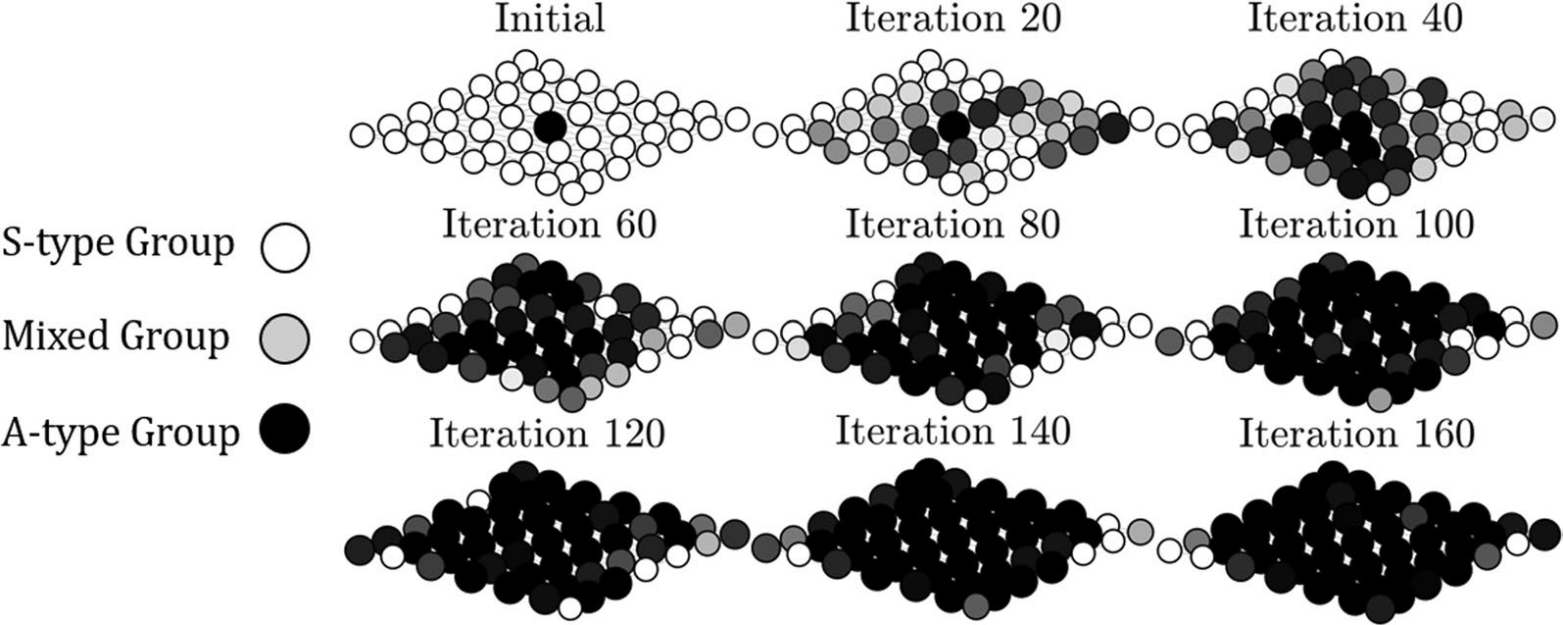
Propagation of the A-type under selective migration when ${R}_{in}=50$. Each panel depicts the $7\times 7$ metapopulation grid. Bubbles indicate the spatial location of groups. The proportion of A-types in a group is represented by hue, with $\pi =0$ (an S-type group) indicated in white and $\pi =1$ (an A-type group) in black. Bubble diameter is proportional to the logarithm of group size. Starting from a single A-type group, the A-type can propagate across the metapopulation by selective migration as depicted, but not by random migration

Sim 1 was initiated with an S-type metapopulation into which one A-type group was placed at the center of a $7\times 7$ grid (see Fig. 3). Selective Migration (SM), Random Migration (RM), and Trait-group Selection (TG) were each applied from this starting point one hundred times with ${R}_{in}=50$ per group per generation for $2\times {10}^{4}$ generations. This was repeated using different values for the number of generations between dispersal events $\left(\Delta g\right)$ to assess the impact of the rate of gene flow between groups might have on the evolutionary process. Results are reported in Table 3. The A-type was always eliminated under RM and TG, as was expected given that whole-group trait altruism is strong. By contrast, the A-type was almost always fixed in the metapopulation under SM. When $\Delta g =100$, for example, the proportion of A-type cells in the metapopulation after $2\times {10}^{4}$ generations averaged over all trials was $\overline{\pi }=0.95$ with a standard deviation of $0.13$, and the A-type was fixed in the metapopulation or nearly so with $\pi >0.95$ in $85/100$ trials. The only exception was when $\Delta g =1$ generation between migration events. In that case, the average proportion of A-type cells in the metapopulation after $2\times {10}^{4}$ generations was only $\overline{\pi }=0.27\pm 0.10$ and the A-type was never fixed in the metapopulation. One trial under SM with $\Delta g=100$ is depicted in Fig. 3.

**Table 3:** Result summaries for metapopulation simulations

Scenario	$\Delta g=100 \mathrm{gens}$	$50 \mathrm{gens}$	$25 \mathrm{gens}$	$1 \mathrm{gen}$
Selective Migration ${R}_{in}=50$	$0.95\pm 0.13$ $85/100$	$0.99\pm 0.01$ $100/100$	$0.99\pm 0.01$ $100/100$	$0.27\pm 0.10$ $0/100$
Random Migration ${R}_{in}=5$	$0.47\pm 0.45$ 28 $/100$	$0.53\pm 0.48$ $46/100$	$0.55\pm 0.46$ $47/100$	$0.41\pm 0.48$ $37/100$
Trait-Group Selection ${R}_{in}=5$	$0.58\pm 0.50$ $58/100$	$0.61\pm 0.49$ $60/100$	$0.64\pm 0.48$ $63/100$	$0.47\pm 0.50$ $46/100$

In the first box $0.95\pm 0.13$ gives the average proportion of A-type cells in the metapopulation after $2\times {10}^{4}$ generations ($\overline{\pi }$) plus/minus one standard deviation taken over 100 simulations. The ratio 85/100 indicates that the A-type was fixed in the metapopulation or nearly so with $\pi >0.95$ in 85 out of 100 trials

**Fig. 4:**
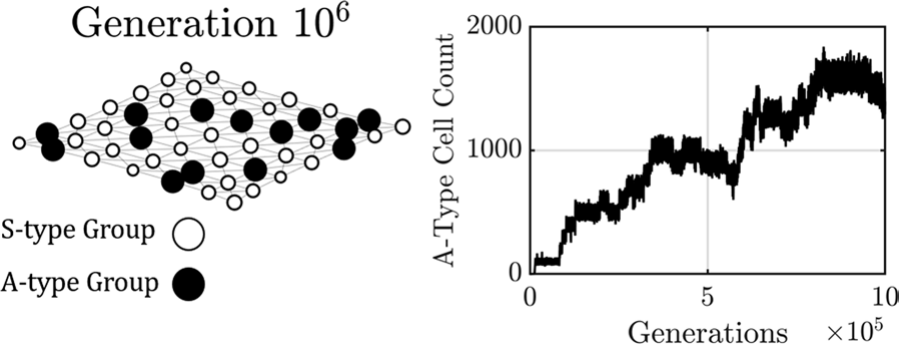
Without dispersal the metapopulation reaches a dynamic equilibrium when ${R}_{in}=5$. The bubble plot depicts the $7\times 7$ grid of the metapopulation after $1\times {10}^{6}$ generations without dispersal. Bubbles indicate the spatial location of groups. The proportion of A-types in a group is represented by hue, with $\pi =0$ (an S-type group) indicated in white and $\pi =1$ (an A-type group) in black. Bubble diameter is proportional to the logarithm of group size. Dots indicate places where stochastic birth–death processes caused a group to go extinct. The line plot shows how the number of A-type cells in the metapopulation evolved over time by mutation and drift starting from zero. The metapopulation gradually approaches a dynamic equilibrium in the number of A-type groups

A single instance of Sim 2 is depicted in Fig. 4. The figure verifies that the metapopulation will tend toward a dynamic equilibrium with a mix of A-type and S-type groups when there is no dispersal and nutrients are severely limited $({R}_{in}=5)$. The simulation was initialized with an S-type metapopulation. Although “switch” mutations were usually eliminated as quickly as they arose (cf. Fig. 1), A-type mutants were occasionally fixed as indicated by the stepwise increase in the proportion of A-types in the metapopulation over the course of ${10}^{6}$ generations. A point of dynamic equilibrium with $14$ A-type groups and approximately $1400$ A-type cells was eventually reached. This is close to the approximate theoretical value of $16$ A-type groups (33% of 49 in accordance with the ratio ${P}_{AS}/{P}_{SA}\approx 2$) and $1600$ A-type cells (since an A-type group has 100 cells at birth–death equilibrium when ${R}_{in}=5$).

Sim 3 was initiated in the same manner as Sim2 (i.e., starting with only S-type cells in the metapopulation) but this time with dispersal by either RM or TG for $2\times {10}^{4}$ generations. Unlike Sim 1, where the initial A-type group was always eliminated under RM and TG, here the A-type reached fixation in the metapopulation or nearly so with $\pi >0.95$ in 28/100 simulations under RM and in 58/100 simulations under TG when $\Delta g=100$ generations (Table 3). The mean value for $\pi$ at the end of these simulations was $\overline{\pi }=0.47\pm 0.45$ under RM and $\overline{\pi }=0.58\pm 0.50$ under TG. Results were similar for other values of $\Delta g$. Note that it is possible for the A-type to be fixed under Random migration even when ${R}_{in}\ge 50$ provided the cost of altruism is reduced to the point that the selection regime is nearly neutral. An S-type metapopulation can transition to an A-type metapopulation by mutation, drift, and random migration when ${R}_{in}=50$ provided $\delta =0.998$ and when ${R}_{in}=500$ provided $\delta =0.9998$, for example. These scenarios are discussed Additional file 1, the latter scenario being illustrated in Additional file 1: Fig. S1.

## Discussion

In this article we investigated the evolution of whole-group trait altruism. It was shown that one A-type group inserted into an S-type metapopulation can proliferate and displace the S-type in all groups under selective migration. This is not satisfactory as an explanation for the evolution of whole-group trait altruism for two reasons. First and foremost, it does not explain the origin of that one group of altruists, which is unlikely to arise when the nutrient influx is relatively large (${R}_{in}=50$). Second, it assumes that only larger groups or those with a larger proportion of altruists can be donors (cf. [36]). This bias opposes within-group selection in a way that shifts the distribution of the ${\pi }_{k}$ toward one, which was necessary given that whole-group altruism is strong when ${R}_{in}\ge 50$. However, it can be argued that selective migration is somewhat artificial and unlikely to occur in a natural setting (cf. [59]). Severe nutrient limitation provides a more plausible explanation for the origin and maintenance of whole-group trait altruism. This is especially true because low nutrient or “oligotrophic” conditions are so common in natural settings in which microbial populations exist (e.g., in the open ocean, in deep subsurface soils, or under the polar ice caps). Nutrient limitation makes it possible for a single A-type mutant to be fixed within an S-type group by drift. Moreover, it makes whole-group trait altruism effectively weak, so that the A-type can propagate and be fixed in the metapopulation under random migration, which is arguably more consistent with what might occur in a natural setting.

Interestingly, our analysis provides an explanation for the evolution of whole-group trait altruism in which drift, rather than positive selection, plays a central role. Severe nutrient limitation makes it possible for an A-type mutant to be fixed by drift within any single S-type group. Once established, an A-type group can effectively re-produce itself under the random migration process. It can be argued that the relatively large size of an A-type group compared to an S-type group, when combined with “weight-of-numbers” migration (i.e., when a migration propagule is proportional to the size of its donor group), increases the inclusive fitness of A-type cells, and that the fixation of the A-type in the metapopulation is therefore by selection. However, the size advantage of A-type groups increases the probability that A-type migrants, when transferred into an S-type recipient group, will be fixed by drift. It likewise increases the probability that S-type migrants, when transferred into an A-type recipient group, will be eliminated by drift. It is therefore possible, as an alternative explanation, to attribute the fixation of whole-group trait altruism to the amplification of drift caused by the joint effect of the metapopulation structure, random migration, and severe nutrient limitation.

The notion of an ecological scaffold provides an explanatory framework for the evolution of traits like that of the A-type in our model. Using simulations, Black et al. [9] aimed to explain how groups of microbes might acquire cooperative traits as a step toward the evolution of a multicellular entity. Their strategy was to show how the environment can give rise to such traits by making individual cells “unwitting participants in a selective process … as part of a larger (collective-level) entity” [9]. Their ecological scaffold consisted of features that generate the familiar spatially segregated population, but also included a dispersal regime that acted to promote groups of cells with a specific average growth rate. They showed that their scaffold can give rise to cells that curtail growth, and thus suffer a reduction in fitness, in return for the opportunity to produce new groups starting from a single nominal “germ” cell drawn randomly from amongst their numbers (cf. [28, 60]).

Does the concept of an ecological scaffold represent an explanatory strategy that is different than kin and multilevel selection theories? One key feature is its emphasis on the role of the environment. Of course, the environment plays a role in determining the probability of all evolutionary outcomes. It is only that environmental conditions are typically consigned to the background or remain unspecified. In Kimura’s diffusion approximation [38], for example, the probability that a mutant is fixed is a function of the effective size (${N}_{e}$) of the single unstructured population in which it is assumed to exist plus a selection coefficient ($s$) that represents the difference between the fitness of the mutant compared to the wildtype. Environmental conditions are largely ignored, although they are arguably implicit in both ${N}_{e}$ and $s$. The environment is similarly relegated to the background in models of kin selection, which often appeal to traits of individual cells such as those that limit dispersal (e.g., via the production of a sticky substance) or that give rise to behavior analogous to kin recognition (e.g., some form of quorum sensing). Yet all models of kin selection and multilevel selection are based on some form of population structured selection. Both theories would seem to fall under the ambit of scaffolding insofar as the requisite conditions for population structured selection are assumed to be imposed by the environment. The notion of an ecological scaffold therefore overlaps with existing theory.

The scaffolding concept nevertheless contributes something more to existing theory by explicitly revealing how the co-occurrence of specific environmental conditions can impact the likelihood of an evolutionary outcome. Here we showed that an A-type group in an otherwise S-type metapopulation cannot persist for long under RM or TG when ${R}_{in}\ge 50$. And although A-type groups can arise in a metapopulation and persist for a time when ${R}_{in}=5$, S-type groups remain dominant in the absence of a dispersal process. Each condition, severe nutrient limitation (${R}_{in}= 5$) and dispersal (RM or TG), is insufficient by itself to support the A-type to fixation in the metapopulation. Instead, it is their interaction that changes the balance between selection and drift in a way that makes the fixation of the A-type in the metapopulation possible.

It is interesting to ask whether the ecological scaffold, as investigated here, can give rise to permanent change in individual traits. It was assumed that A-type and S-type cells can occasionally mutate to one another. An A-type metapopulation will therefore always be vulnerable to any change in the environment that removes one of the components of the scaffold. A sharp increase in the nutrient supply would make it possible for an S-type mutant to proliferate and displace the A-type, for example. However, the subsequent evolution of any trait that reduces the tradeoff between the cost and benefit of whole-group trait altruism might enhance the survivability of the A-type in the absence of the scaffold via a process of endogenization [45, 61].

The term “endogenize” refers to any process by which the ability to resist S-type mutants, initially conferred onto an A-type metapopulation by ecological scaffolding conditions, is “transferred” by subsequent evolutionary change to individual A-type cells. In this way, the externally imposed scaffolding conditions would eventually be unnecessary for the A-type to persist. In our model, once segregation, dispersal, and severe nutrient limitation give rise to the fixation of whole-group trait altruism, there is no evolutionary pressure for the A-type to acquire traits to resist S-types. This makes the A-type vulnerable to S-type resurgence whenever the scaffolding conditions are removed. Imagine, however, that spatial segregation and dispersal were maintained but that the nutrient influx was made to cycle between ${R}_{in}=5$ and ${R}_{in}=50$ slowly enough for the A-type to be repeatedly fixed and eliminated in the metapopulation over macroevolutionary timescales. Mutations that reduce the cost of altruism even slightly might sometimes arise. Periods of larger nutrient influx would favor such A-type mutants. There could follow a process of “persistence selection” [57, 62] whereby some A-type lineages persist across the cycles and eventually accumulate enough reductions in cost to resist the S-type when ${R}_{in}=50$ (cf. [50]). If whole-group trait altruism is based on the production of siderophores, for example, A-type mutants that are more efficient in the uptake of chelated iron would be more resistant to S-type resurgence (cf. [63]). In this way, the production of a public good, which is the hallmark of whole-group trait altruism, might become “endogenized” as a feature of a population that can persist without the ecological scaffold that made its evolution possible.

The evolution of any mechanism that causes altruists to associate more frequently than chance within groups represents another way that an externally imposed scaffolding could become lost over evolutionary time [3, 42]. In the example of costly siderophore production, active positive assortment could increase the frequency of interactions where the siderophores are more available to other A-types than to S-types. However, evolution of assortment by natural selection requires the presence of S-types (because in the absence of S-types, assortment of A-types would be neutral). Thus, an assortative trait must originate and make A-types more fit than S-types *at the time of S-type resurgen*ce, which seems less likely than gradual decreases in the cost of altruism driven by natural section within A-type groups. Interestingly, this implies that endogenization might be more likely to evolve via exaptation [64] than adaptation. The distinction here involves the role of natural selection at the time of trait origination within a group [64, 65]. If reduced cost of altruism evolved gradually by natural selection relative to other A-types and only later conferred resistance to S-type resurgence, such a trait would be, simultaneously, a within-population adaptation and an exaptation for macroevolutionary persistence within the metapopulation. Note that any trait fixed by drift within a group that later confers fitness, at any level, would represent a pre-aptation rather than an exaptation [65]. Future work on the endogenization of altruism should explore the complex interplay of multilevel pre-aptation, exaptation and adaption [65].

## Conclusion

The existence of altruism in nature is theoretically puzzling in view of the fundamentally selfish nature of natural selection. Kin and multilevel selection theories represent two mathematical frameworks that shed light on the problem. Both can be used to show that, although an altruistic individual might suffer a direct deficit in fitness due to its altruistic behavior, it can also receive an indirect benefit that makes its inclusive fitness greater than that of a non-altruist. Hence, kin and multilevel selection theories maintain the view that natural selection is ultimately selfish and largely driven by positive selection. Our simple model provides an alternative way to think about altruism based on the recently introduce concept of an “ecological scaffold”. It shows that altruistic behavior can be favored when the supply of nutrients is severely limited, not necessarily because growth-limitation makes altruists more fit, but because it amplifies drift in a way that favors the propagation of altruistic types in a metapopulation. In addition to nutrient limitation, this requires a metapopulation structure and weight-of-numbers dispersal (e.g., random migration), the three conditions comprising an ecological scaffold for the evolution of whole-group trait altruism. Our model suggests that altruistic and cooperative behaviors may be highly prevalent among microbial populations in low-nutrient “oligotrophic” environments, such as the open ocean, deep subsurface soils, or under the polar ice caps, or anywhere else the conditions of the ecological scaffold are met. Finally, our work implies that altruistic and cooperative behaviors that appear to be endogenous in nature might have originated via multi-level pre-aptation and exaptation facilitated by the presence of an ecological scaffold.

## Methods

Fitness can be defined as the expected contribution that an ancestral individual will make to a descendant population over one ancestor–descendant mapping (e.g., one generation). This can be expressed as the number of offspring an ancestral individual is expected to produce by multiplication plus the probability that the ancestor itself will survive into the descendant population. Using this convention, we define the fitness of the A-type and S-type as follows:

1 $${w}_{A}\left(\pi \right)={\beta }_{A}+\left(1-{D}_{A}(\pi )\right), {w}_{S}\left(\pi \right)={\beta }_{S}+\left(1-{D}_{S}(\pi )\right)$$

Fitness is partially determined by the proportion $\pi$ of A-types in the population via the probability of death, ${D}_{A}\left(\pi \right)$ or ${D}_{S}(\pi )$. It will be assumed that the death rate is the same for both types, ${D}_{A}\left(\pi \right)={D}_{S}(\pi )$, as would be the case in a spatially unstructured population in which the benefit of altruism is conferred to all cells equally. The expected number of offspring produced by an individual, ${\beta }_{A}={c}_{A}/T$ or ${\beta }_{S}={c}_{S}/T$, is the ratio of the expected quantity of nutrient it will consume over one mapping (consumption rate $c$) to the amount of nutrient required to produce one offspring ($T$). For simplicity, we set to $T=1$. The birth rate is an intrinsic property of cells as determined by the presence or absence of $G$. The common death rate $D\left(\pi \right)$, by contrast, is a function of the proportion of A-type cells in the population and is therefore contextual.

### Hard and soft selection

The difference ${w}_{A}-{w}_{S}={\beta }_{A}-{\beta }_{S}<0$ is independent of the composition of the population ($\pi$) and solely attributable to differences in the genes that each type carries. This scenario corresponds to hard selection in favor of the S-type. The situation changes when cells compete for resources. Let ${R}_{in}$ represent the quantity of a growth-limiting nutrient that enters the population at the start of each ancestor–descendant mapping. And let ${n}_{A}$ and ${n}_{S}$ represent the number of A-type and S-type individuals in the population. The “consumption ratio” for the A-type subpopulation is:

2 $$cR\left(\pi \right)= \frac{\delta {n}_{A}}{\delta {n}_{A}+{n}_{S}}\quad\mathrm{ where } \,\delta =\frac{{c}_{A}}{{c}_{S}}<1$$

The A-type subpopulation will consume $cR\left(\pi \right){R}_{in}$ units of nutrient over the next ancestor–descendant mapping, leaving $\left(1-cR\left(\pi \right)\right){R}_{in}$ for the S-type subpopulation. This assumes that all of ${R}_{in}$ is consumed and converted to new cells. In this scenario the difference in fitness depends on $\pi$:

3 $${w}_{A}\left(\pi \right) =\frac{cR\left(\pi \right){R}_{in}}{{n}_{A}}+\left(1-D\left(\pi \right)\right), {w}_{S}\left(\pi \right)=\frac{\left(1-cR\left(\pi \right)\right){R}_{in}}{{n}_{S}}+\left(1-D\left(\pi \right)\right)$$

4 $${w}_{A}\left(\pi \right)-{w}_{S}\left(\pi \right)=-{R}_{in}\left(\frac{1-cR\left(\pi \right)}{{n}_{S}}-\frac{cR\left(\pi \right)}{{n}_{A}}\right)=-{R}_{in}\left(\frac{1-\delta }{\delta {n}_{A}+{n}_{S}}\right)<0$$

The A-type is still less fit that the S-type, just as it was under hard selection. Now, however, the difference is a decreasing function of $\pi$. This scenario corresponds to soft selection in favor of the S-type.

**Fig. 5:**
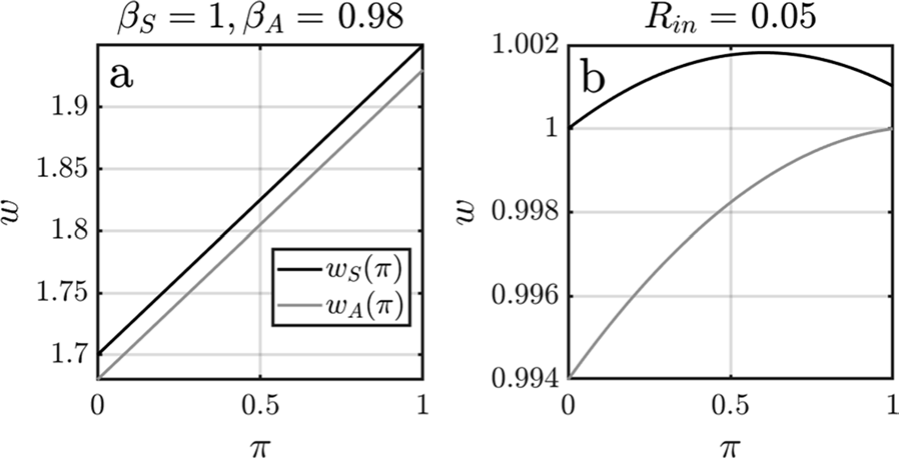
An illustration of the contrast between hard (**a**) and soft (**b**) selection using hypothetical values for model parameters. The fitness of both the altruistic A-type and selfish S-type are assumed to increase with the proportion $\pi$ of A-types in the population. However, **a** whereas the fitness differential ${w}_{S}\left(\pi \right)-{w}_{A}\left(\pi \right)$ remains constant in the absence of nutrient limitation (Eq. 1), **b** it gets smaller as $\pi \to 1$ when nutrients are limited

The contrast between hard and soft selection is illustrated in Fig. 5. Figure 5a illustrates Eq. 1, where the fitness of both types increases with $\pi$ with a constant difference between them. The mean fitness $\overline{w }=\pi {w}_{A}+\left(1-\pi \right){w}_{S}$ under this scenario is always greater than one, reflecting unlimited growth. Figure 5b illustrates Eq. 3, where the difference in fitness decreases with $\pi$. In this case the mean fitness is $\overline{w }=1$ consistent with a population at birth–death equilibrium (i.e., when ${n}_{A}+{n}_{S}={R}_{in}/D(\pi )$).

### The relative cost and benefit of whole-group trait altruism

Let ${z}_{i}\in \left\{\mathrm{0,1}\right\}$ for $i\in \{A,S\}$ be an indicator for the presence (${z}_{A}=1$, A-type) or absence (${z}_{S}=0$, S-type) of a functional copy of the gene complex $G$ for the altruistic behavior. The relationship between fitness and genotype conditioned on the proportion of A-types in the population is a simple linear function:

5 $${w}_{i}\left(\pi \right)={w}_{S}\left(\pi \right)+\left({w}_{A}\left(\pi \right)-{w}_{S}\left(\pi \right)\right)z_i$$

The slope ${w}_{A}\left(\pi \right)-{w}_{S}\left(\pi \right)$ gives the relative fitness of an A-type cell compared to an S-type. This can be equated to the inclusive fitness of an A-type cell if inclusive fitness is construed as a relative quantity (i.e., A-type fitness relative to S-type fitness). This slope is always negative, so the benefit of whole-group trait altruism never outweighs the cost. This is precisely because the benefit is conferred to all cells in the population equally. It follows that an A-type mutant that arises in a single S-type population cannot be fixed by positive selection.

It is instructive to consider conditions under which the A-type *can* be fixed by selection. The uptake of public goods often requires specific receptors, and these may vary between selfish and altruistic types. When altruism is based on the production of siderophores, for example, a selfish type might have less capacity for the uptake of publicly available chelated iron compared to the altruistic type [63]. It is therefore plausible to model the S-type as receiving only a portion $\mathrm{\alpha }\in \left(\mathrm{0,1}\right)$ of the benefit of altruism. At this point we must define a functional form for the probability of death per cell per ancestor–descendant mapping. For simplicity, let us suppose it is a linear function of $\pi$ with values ranging between ${D}_{min}$ in an A-type population (when $\pi =1$) to ${D}_{max}$ in an S-type population (when $\pi =0$). The impact of $\alpha$ might then be modelled as follows:

6 $${D}_{A}\left(\pi \right)={D}_{max}+\pi \left({D}_{min}-{D}_{max}\right), {D}_{S}\left(\pi \right)={D}_{max}+\alpha \pi \left({D}_{min}-{D}_{max}\right)$$

The difference in the fitness of the two types now depends on $\alpha$:

7 $${w}_{A}\left(\pi \right)-{w}_{S}\left(\pi \right)=\pi \left(1-\alpha \right)\left({D}_{max}-{D}_{min}\right)-{R}_{in}\left(\frac{1-\delta }{\delta {n}_{A}+{n}_{S}}\right)$$

The first term on the RHS of Eq. 7 corresponds to the benefit that accrues to a focal A-type cell above that accrued by an S-type cell in the same population. The second term corresponds to the cost of altruism under soft selection. The A-type will be favored by selection whenever $\alpha$ makes Eq. 7 positive. This demonstrates how intrinsic properties of cells can alter the balance between the cost and benefit of the production of a public good. Note, however, that in a scenario in which $\alpha$ is small enough to give the A-type a fitness advantage, the A-type is no longer an altruist. We therefore proceed by assuming $\alpha =1$ when whole-trait group altruism comes at a cost with no relative benefit.

### The multilevel selection perspective

Multilevel selection (MLS) theory accounts for evolution in a population of groups in which selection can act within groups and between groups at the same time. Two scenarios are usually considered. In the first (MLS1, 29), the fitness of an individual is assumed to be the sum of a component attributed to a character state (genotype or phenotype) plus a contextual component that is a function of the mean character state of the group in which it exists (cf. contextual analysis, [66]). The classical scenario occurs when the mean fitness of a group is correlated with the proportion of altruists it contains. In the second (MLS2, [32]), a group is assigned a fitness that is independent of the mean fitness of the individuals it contains. Instead, the fitness of a group reflects its ability to reproduce as a group. Groups are therefore equated to Darwinian individuals and the collection of groups to a Darwinian population [67]. In practice, MLS2 is often artificially imposed (e.g., [68, 69]), although it is applicable to certain natural systems or processes (e.g., an evolutionary transition in individuality, [33]). Only MLS1 is considered in this article.

The product of the mean fitness $(\overline{w })$ and the change in the mean character state ($\Delta \overline{z }$) of all individuals in a metapopulation over one ancestor–descendant mapping can be expressed using the Price equation for MLS1 [33, 70, 71]:

8 $$\overline{w }\Delta \overline{z }=\mathrm{Cov}\left({\overline{w} }_{k},{\overline{z} }_{k}\right)+\mathrm{E}\left(\mathrm{Cov}\left({w}_{ik},{z}_{ik}\right)\right)$$

For simplicity, we assume that offspring are identical to parents. The parameters ${z}_{ik}$ and ${w}_{ik}$ represent the character state and fitness of the ${i}\text{th}$ individual in the ${k}\text{th}$ group and ${\overline{z} }_{k}$ and ${\overline{w} }_{k}$ represent the mean character state and mean fitness of all individuals in the ${k}\text{th}$ group. The first term on the RHS of Eq. 8 accounts for change over one ancestor–descendant mapping caused by differences in growth rate (${\overline{w} }_{k}$) in accordance with differences in group composition (${\overline{z} }_{k}$). The second term accounts for change due to individual-level selection within groups. It is important to note that Eq. 8 does not account for processes by which groups exchange individuals (but see [72]). In the absence of a dispersal process, change in the mean character state over one mapping is therefore solely determined by the relative magnitude of the differential growth of groups compared to within-group selection.

The character state in our model is the indicator for the presence or absence of the gene for altruism, ${z}_{ik}\in \left\{\mathrm{0,1}\right\}$, where an A-type corresponds to ${z}_{Ak}=1$. The mean character state of a group is therefore ${\overline{z} }_{k}={\pi }_{k}$ and the mean character state of the metapopulation is $\overline{z }=\pi$. Using these identities, Eq. 8 can be rewritten as follows (Additional file 1):

9 $$\overline{w }\Delta \pi =\left({D}_{max}-{D}_{min}\right)\mathrm{Var}\left({\pi }_{k}\right)+\mathrm{E}\left(\left({w}_{A}\left({\pi }_{k}\right)-{w}_{S}\left({\pi }_{k}\right)\right){\pi }_{k}\left(1-{\pi }_{k}\right) \right)$$

The first term on the RHS of Eq. 9 accounts for the growth advantage enjoyed by groups with a larger proportion of A-type cells. Note that, unlike the case of a single population where all cells suffer the same probability of death, here there is a group effect proportional to the difference between the death rate within an S-type group (${D}_{max}$) compared to an A-type group (${D}_{min}$). The second term in Eq. 9 accounts for the cost of whole-group trait altruism within groups and contains the difference ${w}_{A}\left({\pi }_{k}\right)-{w}_{S}\left({\pi }_{k}\right)<0$.

### The role of dispersal

When resources are limited, all groups in the metapopulation will tend toward birth–death equilibrium with mean fitness ${\overline{w} }_{k}\approx 1$, the main exception being the short periods of time during which a mutant is in the process of being fixed or eliminated (i.e., assuming a simple “genetic switch” model). Under this condition, $\overline{w }\Delta \pi$ is approximately zero apart from stochastic fluctuations (cf. Fig. 4). Directional change in $\pi$ toward one is possible only if some form of dispersal is imposed that gives larger groups (those with ${\pi }_{k}$ closer to one) a chance to replace smaller groups (those with ${\pi }_{k}$ closer to zero).

This last statement alludes to what we call weight-of-numbers dispersal whereby the expected size of a dispersal “propagule” [73] composed of individuals from one group that are to be transferred into one or more other groups, is proportional ($p$) to the size of the group from which it was drawn. Consider trait-group selection, where groups are episodically collected into a common pool from which new groups are randomly drawn. In this case a dispersal propagule is the entire group ($p=1$). Groups of altruists, which are larger than groups of selfish types, contribute more individuals to the common pool. This gives the A-type an advantage in numbers that makes it possible for the A-type to increase in frequency in the metapopulation despite its fitness deficit within groups.

### Dispersal by migration

Curiously, MLS1 is almost exclusively depicted in the literature as some form of trait-group selection. Consider instead dispersal by migration. For simplicity, let us assume that groups are homogenous with either $\pi =0$ (an S-type group) or $\pi =1$ (an A-type group). Assuming groups at birth–death equilibrium, the proportions ${\pi }_{AS}$ (when an A-type group is the donor) and ${\pi }_{SA}$ (when an S-type group is the donor) are determined by the ratio of the size ${N}_{A}={R}_{in}{/D}_{min}$ of an A-group compared to the size ${N}_{S}={R}_{in}{/D}_{max}$ of an S-type group:

10 $${\pi }_{AS}=\frac{{pN}_{A}}{{pN}_{A}+{N}_{S}}=\frac{p}{p+\frac{{D}_{min}}{{D}_{max}}}, {\pi }_{SA}=\frac{{N}_{A}}{{N}_{A}+p{N}_{S}}=\frac{1}{1+p\times \frac{{D}_{min}}{{D}_{max}}}$$

The probability that a propagule drawn from an A-type donor group is fixed by drift in an S-type recipient group is an increasing function of ${\pi }_{AS}$. Similarly, the probability that a propagule drawn from an S-type donor group is eliminated by drift in an A-type recipient group is an increasing function of ${\pi }_{SA}.$ Both probabilities therefore increase with the ratio ${N}_{A}/{N}_{S}={D}_{max}/{D}_{min}$. This shows how the advantage in size enjoyed by A-type groups, when combined with the migration process, makes it possible for the A-type to proliferate across the metapopulation by drift despite its fitness deficit within groups.

### The impact of nutrient limitation on the strength of whole-group trait altruism

Variations between theoretical models of altruism are determined in part by the sign of the hypothetical change in the fitness of a focal S-type individual if it were to switch to an A-type [41]. Assuming a group of constant size ${N=n}_{A}+{n}_{S}$, the proportion of A-type cells is $\pi ={n}_{A}/N$ before the S-to-A switch and ${\pi }^{^{\prime}}=\left({n}_{A}+1\right)/N$ after the switch. A class I fitness structure is indicated when a focal S-type, if it were to switch to an A-type, would lose fitness, ${{w}_{A}\left({\pi }^{^{\prime}}\right)-w}_{S}\left(\pi \right)<0$. A class II fitness structure is indicated when the focal S-type would gain fitness, ${{w}_{A}\left({\pi }^{^{\prime}}\right)-w}_{S}\left(\pi \right)>0$ [41]. These conditions correspond to strong and weak altruism, respectively, as defined elsewhere in the literature (e.g., [33], pp 192–193).

The fitness structure under the present model of whole-group trait altruism (Eq. 3) depends in part on the level of nutrient influx. Consider the change in the fitness of a focal S-type that switches to an A-type given by the following expression (Additional file 1):

11 $${{w}_{A}\left({\pi }^{^{\prime}}\right)-w}_{S}\left(\pi \right)=-\frac{\left(\delta {n}_{A}+{n}_{S}-1\right)\left(1-\delta \right){R}_{in}}{\left(\delta {n}_{A}+{n}_{S}\right)\left(\delta \left({n}_{A}+1\right)+{n}_{S}-1\right)}+\frac{{D}_{max}-{D}_{min}}{N}$$

**Fig. 6:**
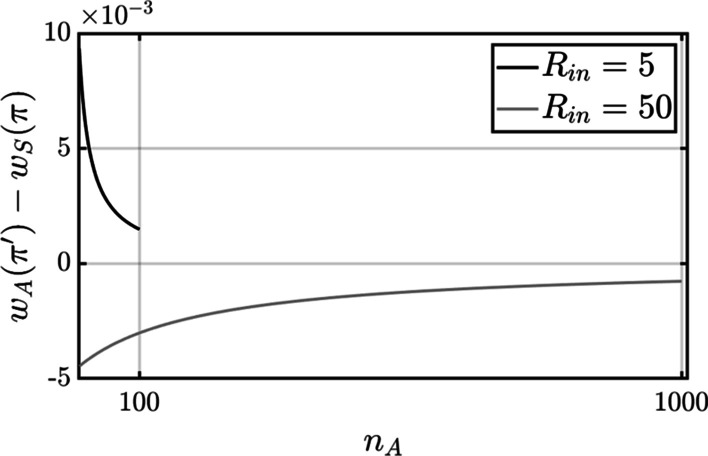
The effect of nutrient supply on the strength of altruism. The difference ${{w}_{A}\left({\pi }^{\mathrm{^{\prime}}}\right)-w}_{S}\left(\pi \right)$ represents the change in the fitness of a focal S-type if it were to convert to an A-type. This difference is negative under the larger nutrient supply ${R}_{in}=50$ (when ${N}_{cap}\left(\pi =1\right)=1000$ A-type cells), indicating that whole-group trait altruism is strong. But it is positive under the smaller nutrient supply ${R}_{in}=5$ (when ${N}_{cap}\left(\pi =1\right)=100$ A-type cells). The reduction in the influx of nutrient therefore causes a shift in the fitness structure that makes whole-group trait altruism effectively weak

The first term on the RHS of Eq. 11 accounts for the loss in fitness the focal S-type suffers due to the reduction in its rate of consumption when it switches to an A-type. The second term accounts for the corresponding gain in fitness caused by the incremental decrease in the common death rate when the proportion of A-type cells increases by ${\pi }^{^{\prime}}-\pi =1/N$. The cost is proportional to ${R}_{in}$ and therefore decreases as ${R}_{in}$ approaches zero. The benefit is inversely proportional to the size of the group and therefore increases as ${R}_{in}$ approaches zero (since $N\propto {R}_{in}$). This opposition in the direction of change suggests that there is a level of nutrient influx at which Eq. 11 shifts from negative to positive. This shows that, although whole-group trait is strong, it can be effectively weak under severe nutrient limitation. The shift in sign is verified by the plots of Eq. 11 with ${R}_{in}=5$ 0 and ${R}_{in}=5$ shown in Fig. 6.

### Single population simulations

The single population model was implemented in MATLAB (R2021a) using custom scripts. Each model iteration corresponding to one ancestor–descendant mapping and included the following steps.

1. The proportion $\pi \left(t\right)$ of A-types in the population was computed, where $t$ is time in generations. This was used to determine the consumption ratio $cR\left(\pi ,t\right)$.
2. The number of descendants produced by each ancestral A-type and S-type cell was determined by a Poisson random variable (one draw for each cell) with the following expected values (where ${n}_{A}$ and ${n}_{S}$ represent the number of cells of each type in the population):
  $$\mathrm{E}\left({w}_{A}\left(\pi ,t\right)\right) =\frac{cR\left(\pi ,t\right){R}_{in}}{{n}_{A}}, \mathrm{E}\left({w}_{S}\left(\pi ,t\right)\right) =\frac{\left(1-cR\left(\pi ,t\right)\right){R}_{in}}{{n}_{S}}$$
3. Each ancestral A-type and S-type cell was then culled by drawing a Bernoulli random variable, a value of 1 corresponding to cell death occurring with probability:
  $$D\left(\pi \right)={D}_{max}+\pi \left({D}_{min}-{D}_{max}\right)$$

It is possible for all ancestral cells to produce no offspring and then to all die, resulting in the extinction of the population. However, simulations were used to verify that this outcome very rarely occurs. In Fig. 1, for example, the population persisted for ${10 }^{7}$ generations without going extinct.

4) Mutations that switch cell type were then accounted for using a Bernoulli random variable with probability ${P}_{mut}={10 }^{-6}$ per cell per generation, a value of 1 corresponding to a switch in cell type.

### Probability estimates in Table 1

The fate of an A-type mutant in an S-type population was determined by converting one cell in an S-type population of size ${n}_{S}=\mathrm{round}\left({R}_{in}/{D}_{max}\right)$ (i.e., at birth–death equilibrium) to an A-type and then running the population model until the A-type was either fixed or eliminated. This was repeated ${10}^{5}$ times to estimate the probability of A-type fixation. The fate of an S-type mutant in an A-type population was estimated in a similar way starting with an A-type population of size ${n}_{A}=\mathrm{round}\left({R}_{in}/{D}_{min}\right)$ in which one cell was converted to an S-type.

### The ratio of switching probabilities ${{\varvec{P}}}_{{\varvec{A}}{\varvec{S}}}/{{\varvec{P}}}_{{\varvec{S}}{\varvec{A}}}$

The probability that a population or group of one cell type will transition to a population of the opposite type when ${R}_{in}=5$ or $50$ was estimated using values reported in Table 1:

$$\frac{{P}_{AS}}{{P}_{SA}}=\frac{{D}_{max}}{{D}_{min}}\times \frac{{P}_{S}^{fix}\left(\delta =0.98,{R}_{in}=5\right)}{{P}_{A}^{fix}\left(\delta =0.98,{R}_{in}=5\right)}\approx 6\times \frac{{1.6\times 10}^{-2}}{{4.4\times 10}^{-2}}\approx 2.18$$

$$\frac{{P}_{AS}}{{P}_{SA}}=\frac{{D}_{max}}{{D}_{min}}\times \frac{{P}_{S}^{fix}\left(\delta =0.98,{R}_{in}=50\right)}{{P}_{A}^{fix}\left(\delta =0.98,{R}_{in}=50\right)}\approx 6\times \frac{{1.6\times 10}^{-2}}{{4.0\times 10}^{-5}}\approx 2400$$

### The probability of A-type fixation in Fig. 2

The fate of the A-type in a mixed population was determined by starting with ${n}_{A}+{n}_{S}=\mathrm{round}\left({R}_{in}/D\left(\pi \right)\right)$ cells in total, the number at birth–death equilibrium for the given proportion $\pi$, and then running the population until the A-type was either fixed or eliminated. This was repeated ${10}^{5}$ times to estimate the probability of A-type fixation for values of $\pi \in \left\{0.05, 0.10,\dots , 0.95\right\}$ and with ${R}_{in}\in \left\{5, 50\right\}$.

### Metapopulation simulations

Simulations were conducted in a metapopulation consisting of $N=49$ groups spatially arranged in $7\times 7$ grid. Three forms of dispersal were considered, selective migration (SM), random migration (RM), and trait-group Selection (TG). The interval between dispersal events in generations included values of $\Delta g\in \left\{1, 25, 50, 100\right\}$. The probability of a “switch” mutation was set to ${P}_{mut}={10}^{-6}$ per cell per generation, each generation corresponding to one iteration of the stochastic population model.

Each iteration of the metapopulation model consisted of implementing the chosen dispersal process and then running the population model within each group independently for $\Delta g$ generations. All simulations were conducted with a “viscosity” parameter set to $v=1$, which indicates that migrations could only occur between neighboring groups, a group having three, five, or eight neighbors, depending on whether it was on the corner, edge, or interior of the $7\times 7$ grid. Migrations would have occurred between any pair of groups regardless of the distance between them if the viscosity parameter was set to $v=0$. Simulations that were conducted in this way (but not reported) had very similar results to the ones reported.

### Random migration

One round of random migrations was implemented as follows:

1. A donor group was randomly selected from among groups that have not yet played the role of donor or recipient during the current model iteration.
2. A recipient group was selected from amongst the donor’s neighbors (i.e., with $v=1$). A migration event would only occur if at least one neighbor had not yet played the role of donor or recipient.
3. A proportion $p$ was drawn from a beta distribution with shape parameters $\left(\alpha , \beta \right)=\left(5, 15\right)$ and expected value $\mathrm{E}\left(p\right)=0.25$. This proportion of A-type and S-type cells were then transferred from the donor group into the recipient group.
4. Ten random migration events at most were implemented with each model iteration.

### Selective migration

One round of selective migrations was implemented as follows:

1. The ten largest groups were identified as candidate donors. Since A-type groups are larger than S-type groups, this tended to preclude the selection of S-type groups as donors.
2. A donor group was selected from amongst the ten candidates that have not yet played the role of donor during the current model iteration.
3. A recipient group was selected from amongst the donor’s neighbors, excluding other candidate donors. A migration event would occur only if at least one such neighbor existed.
4. A proportion $p$ was drawn from a beta distribution with expected value $\mathrm{E}\left(p\right)=0.25$. This proportion of A-type and S-type cells were then transferred from the donor group into the recipient group.
5. Ten selective migration events at most were implemented with each model iteration.

### Trait-group selection

The process of pooling and redistribution was mimicked by assigning a number between 1 and 49 drawn from a discrete uniform distribution to each cell in the metapopulation. A new set of 46 groups was then assembled according to the numbers drawn. Note that this process homogenizes groups with respect to the proportion of A-types they contain, but also potentially gives the A-type a foothold in all 49 groups (i.e., if most groups were S-type). Under all three forms of dispersal there followed the implementation of the stochastic population model to each group independently for $\Delta g$ generations. See Table 4 for a list of model parameters.

**Table 4:** Model parameters and notation

Parameter	Interpretation	Values used
${R}_{in}$	Nutrient influx per group per generation	$5\, \mathrm{or}\, 50$
$\delta$	The fitness deficit suffered by A-type cells	$0.98\, \mathrm{or}\, 1.00$
${D}_{min}$	The probability of death per cell per generation in an A-type population	$0.05$
${D}_{max}$	The probability of death per cell per generation in an S-type population	$0.30$
${P}_{mut}$	The probability of a “switch” mutation per cell per generation	${10 }^{-6}$
$\Delta g$	Number of generations between sets of dispersal events	$1, 25, 50, 100$
$p$	The proportion of cells drawn from a donor group for form a migration propagule	$0.25$
${N}_{A}={N}_{cap}(\pi =1)$	The number of cells in an A-type group at birth–death equilibrium	${R}_{in}{/D}_{min}$
${N}_{S}={N}_{cap}(\pi =0)$	The number of cells in an S-type group at birth–death equilibrium	${R}_{in}{/D}_{max}$

Notation	Interpretation
${w}_{A}\left(\pi \right)$	The fitness of an A-type cell in a population in which the proportion of A-type cells is $\pi$
${w}_{S}\left(\pi \right)$	The fitness of an S-type cell in a population in which the proportion of A-type cells is $\pi$
$cR(\pi )$	The proportion of nutrient influx ${R}_{in}$ consumed by the subpopulation of A-type cells within any population or group
${P}_{AS}$	Probability that an A-type population will transition to an S-type population by selection following an A-to-S-type mutation in a single A-type cell
${P}_{SA}$	Probability that an S-type population will transition to an A-type population by drift following an S-to-A-type mutation in a single S-type cell
${\pi }_{AS}$	The expected proportion of A-type cells in a recipient S-type group after it has received a migration propagule from an A-type donor
${\pi }_{SA}$	The expected proportion of A-type cells in a recipient A-type group after it has received a migration propagule from an S-type donor
${W}_{A}$	The nominal fitness of an A-type group in a metapopulation with dispersal by migration
${W}_{S}$	The nominal fitness of an S-type group in a metapopulation with dispersal by migration
${F}_{A}$	The proportion of A-type groups in a metapopulation
${F}_{S}$	The proportion of S-type groups in a metapopulation

## Supplementary Information

**Additional file 1.** This file includes derivations for eq. 9 (The Price Equation for MLS1) and eq. 11 (Weak vs Strong Altruism) and results of an additional simulation showning A-type fixation with a different set of model parameters.

## Data Availability

All simulations and calculations were implemented in MATLAB version R2021a under license number 861043 for academic use using custom scripts. Scripts are available on GitHub, https://doi.org/10.5281/zenodo.7231567.

## Abbreviations

MLS: Multilevel selection
MLS1: Multilevel selection 1
MLS2: Multilevel selection 2
SM: Selective migration
RM: Random migration
TG: Trait-group selection

## Footnotes

1 It has been said that “an altruistic behavior will be favored by selection so long as the fitness cost to the actor is offset by a sufficient amount of benefit to sufficiently closely related recipients” [4]. In fact, individuals need not be related by descent [3, 15–17], especially among microbes due to their ability to acquire genes by horizontal transfer [18]. It is only that the benefit of altruism must preferentially accrue to other altruists and that the kin of an altruistic individual are often more likely to be altruists themselves.

2 Theoretical models have been used to demonstrated how the advantage that altruism gains from limited dispersal is exactly offset by the impact of kin competition [23]. However, simulations [24] and empirical studies [25] have since shown that there are conditions under which population viscosity can support altruism.

3 This definition takes the perspective of a focal non-altruist. Wilson [40], by contrast, takes the perspective of a focal altruist, defining strong altruism to be the case where the altruist suffers an absolute reduction in fitness because of its altruistic behavior and weak altruism to be the case where the altruist suffers only a relative reduction in fitness. Both perspectives are isomorphic with the Class I and Class II fitness structures for altruism as defined by Kerr et al. [41].

4 Strong altruism can also be supported in other ways. For example, Fletcher and Zwick [43] explores conditions under which strong altruism might evolve in randomly formed groups. Similarly, Simon et al. [44] provides a general model of group selection under which cooperative behavior can evolve without positive assortment.

5 Empirical and simulation studies that highlight the importance of environmental conditions, including nutrient limitation, on the evolution of cooperation and/or altruism do exist (e.g., [9, 25, 48–50]). Such studies are in accordance with the notion that an ecological scaffold can alter fitness structures in a way that supports the evolution of cooperative behavior.

6 Selfishness is not necessarily the natural starting point of a modeled scenario. For example, selfishness can evolve in a non-selfish population from an individual mutant that has lost the ability to produce a costly public good [28, 51, 52].

7 Based on Kimura’s equation for the probability of fixation [38].

8 We are following Doolittle and Inkpen [57], who make a biologically informed distinction between scenarios of reproduction, where there is a direct association between parent and offspring, and scenarios of “re-production”, where entities such as communities of microbes have no such association but can nevertheless be “created again”.

9 The terms “re-production” and “nominal fitness” are only used to help explain how the proportion of A-type individuals in the metapopulation can increase via the fixation (by drift) of A-type migration propagules within S-type recipient groups. We make no ontological claim about whether the groups can be construed as Darwinian individuals [32] that satisfy the three ingredients of Lewontin’s recipe [58].
