# Supplementary material for: The role of the ecological scaffold in the origin and maintenance of whole-group trait altruism in microbial populations
Source: BMC Ecol Evol. 2023 Apr 12;23:11. doi: 10.1186/s12862-023-02112-2 (PMC10100367; doi:10.1186/s12862-023-02112-2)
Supplement: Supplementary file 1 — Additional file 1. This file includes derivations for eq. 9 (The Price Equation for MLS1) and eq. 11 (Weak vs Strong Altruism) and results of an additional simulation showning A-type fixation with a different set of model parameters. [file 12862_2023_2112_MOESM1_ESM.pdf]

Supplementary

The Price Equation for MLS1 (eq. 9)

The coefficient of regression $b(y,x)$ of y onto x is the ratio $b(y,x) = \mathrm{Cov}(y,x)/\mathrm{Var}(x)$. Hence:

$$\mathrm{Cov}(\overline{w}_k, \pi_k) = b(\overline{w}_k, \pi_k)\mathrm{Var}(\pi_k)$$

It will be assumed that the kth group has an arbitrary number of A-type (n_{Ak}) and S-type (n_{Sk}) cells, not necessarily the number that would be observed at birth-death equilibrium.

Calculating:

$$\overline{w}_k = w_S(\pi_k) + \big(w_A(\pi_k) - w_S(\pi_k)\big)\pi_k$$

$$\overline{w}_k = R_{in}\frac{1 - cR(\pi_k)}{n_{Sk}} + \big(1 - D(\pi_k)\big) - R_{in}\left(\frac{1-\delta}{\delta n_{Ak} + n_{Sk}}\right)\pi_k$$

$$\overline{w}_k = R_{in}\frac{1}{\delta n_{Ak} + n_{Sk}} + \big(1 - D(\pi_k)\big) - R_{in}\left(\frac{1-\delta}{\delta n_{Ak} + n_{Sk}}\right)\frac{n_{Ak}}{n_{Ak} + n_{Sk}}$$

$$\overline{w}_k = R_{in}\left(\frac{n_{Ak} + n_{Sk}}{(\delta n_{Ak} + n_{Sk})(n_{Ak} + n_{Sk})} - \frac{(1-\delta)n_{Ak}}{(\delta n_{Ak} + n_{Sk})(n_{Ak} + n_{Sk})}\right) + \big(1 - D(\pi_k)\big)$$

$$\overline{w}_k = R_{in}\left(\frac{\delta n_{Ak} + n_{Sk}}{(\delta n_{Ak} + n_{Sk})(n_{Ak} + n_{Sk})}\right) + \big(1 - D(\pi_k)\big)$$

$$\overline{w}_k = R_{in}\left(\frac{1}{n_{Ak} + n_{Sk}}\right) + 1 - D_{max} + (D_{max} - D_{min})\pi_k$$

The coefficient of regression of $y = \overline{w}_k$ onto $x = \pi_k$ is therefore:

$$b(\overline{w}_k, \pi_k) = D_{max} - D_{min} > 0$$

Similarly:

$$\mathrm{Cov}(w_{ik}, z_{ik}) = b(w_{ik}, z_{ik}|\pi_k)\mathrm{Var}(z_{ik}|\pi_k) \text{ where } \mathrm{Var}(z_{ik}|\pi_k) = \pi_k(1 - \pi_k)$$

Calculating:

$$w_{ik} = w_S(\pi_k) + \big(w_A(\pi_k) - w_S(\pi_k)\big)z_{ik}$$

The coefficient of regression of $y = w_{ik}$ onto $x = z_{ik}$ is therefore:

$$b(w_{ik}, z_{ik}|\pi_k) = w_A(\pi_k) - w_S(\pi_k) < 0$$

It follows that:

$$\overline{w}\Delta\pi = \mathrm{Cov}(\overline{w}_k, \pi) + \mathrm{E}\big(\mathrm{Cov}(w_{ik}, z_{ik})\big)$$

$= b(\overline{w}_k, \pi_k)\mathrm{Var}(\pi_k) + \mathrm{E}(b(w_{ik}, z_{ik}|\pi_k)\pi_k(1-\pi_k))$

$= (D_{max} - D_{min})\mathrm{Var}(\pi_k) + \mathrm{E}\left(\left(w_A(\pi_k) - w_S(\pi_k)\right)\pi_k(1-\pi_k)\right)$

Weak vs Strong Altruism (re. eq. 11)

The common death rate was assumed to be a linear function of the proportion of A-types in the population:

$$D(n_A, n_S) = D_{max} + (D_{min} - D_{max})\frac{n_A}{n_A + n_S}$$

The fitness of an individual "focal" S-type as a function of the number n_A of A-types in the population is:

$$w_S(n_A, n_S) = \frac{R_{in}}{\delta n_A + n_S} + \left(1 - D_{max} + (D_{max} - D_{min})\frac{n_A}{N}\right)$$

The fitness of the same individual, if it were to convert to an A-type (i.e., assuming $n_A + n_S = N$ is constant), is:

$$w_A(n_A + 1, n_S - 1) = \frac{\delta R_{in}}{\delta(n_A + 1) + n_S - 1} + \left(1 - D_{max} + (D_{max} - D_{min})\frac{n_A + 1}{N}\right)$$

The change in the fitness of the focal S-type is the difference:

$$w_A(\pi') - w_S(\pi) = -\frac{(\delta n_A + n_S - 1)(1-\delta)R_{in}}{(\delta(n_A + 1) + n_S - 1)(\delta n_A + n_S)} + \frac{D_{max} - D_{min}}{N}$$

$$\text{where } \pi = \frac{n_A}{N} \text{ and } \pi' = \frac{n_A + 1}{N}$$

Calculating:

$$w_A(\pi') - w_S(\pi) = -\frac{(1-\delta)R_{in}}{\delta n_A + n_S} \times \frac{\delta n_A + n_S - 1}{\delta(n_A + 1) + n_S - 1} + \frac{D_{max} - D_{min}}{N}$$

$$w_A(\pi') - w_S(\pi) = \left(w_A(\pi) - w_S(\pi)\right)\frac{\delta n_A + n_S - 1}{\delta(n_A + 1) + n_S - 1} + \frac{D_{max} - D_{min}}{N}$$

A-type fixation under higher nutrient influx.

Using simulations, it was shown that an S-type metapopulation can transition to an A-type metapopulation by mutation and drift provided the selective regime is nearly neutral. Such a regime can arise when nutrients are severely limited (e.g., $R_{in} = 5$ when $\delta = 0.98$) but can also arise when the cost of altruism is very small. When $\delta = 0.998$, for example, an S-type metapopulation can transition to an A-type metapopulation even when $R_{in} = 50$. Using simulations, it was determined that the probability that an A-type mutant is fixed in an S-type population is approximately 5.7×10^{-3} when $\delta = 0.998$ and $R_{in} =$

50, whereas the probability that an S-type mutant is fixed in an A-type population is approximately 6.3×10^{-3}. The ratio P_{AS}/P_{SA} is therefore $6 \times 3.3/5.7 = 6.63$ or approximately 7 assuming $D_{max}/D_{min} = 6$. This means that, in the absence of dispersal, an S-type metapopulation will evolve toward a dynamic equilibrium with approximately $49/7 = 7$ A-type groups and 42 S-type groups and suggests that the introduction of random dispersal can lead to the fixation of the A-type in the metapopulation, just as it did when $\delta = 0.98$ and $R_{in} = 5$.

The larger R_{in}, the smaller δ must be for the A-type to have a chance for become fixed in the metapopulation. When $R_{in} = 500$, for example, an A-type and S-type population will have $500/D_{min} = 10{,}000$ cells and $500/D_{max} = 1{,}666$ cells at birth-death equilibrium, respectively. In this case, an A-type mutant has a reasonable chance to reach fixation in an S-type group only when delta is very close to one. When $\delta = 0.9998$, for example, simulations indicate that the probability that an A-type mutant is fixed in an S-type population is approximately 7.0×10^{-4}, and the probability that an S-type mutant in an A-type population is fixed is approximately 2.0×10^{-4}. Interestingly, in this case, the A-type mutant is more likely to be fixed by drift than an S-type is to be fixed by selection. This is because selection is nearly neutral, and the probability of fixation under neutrality is $1/1666 = 6 \times 10^{-4}$ for an A-type mutant in the smaller S-type population but only $1/10{,}000 = 1 \times 10^{-4}$ for an S-type mutant in the larger A-type population. The ratio P_{AS}/P_{SA} is now $6 \times 2/7 = 1.21$, which suggests that an S-type metapopulation can transition to an A-type metapopulation by mutation and drift, like the $\delta = 0.98$ and $R_{in} = 5$ scenario. This possibility is verified by the simulation depicted in **Fig. S1**.

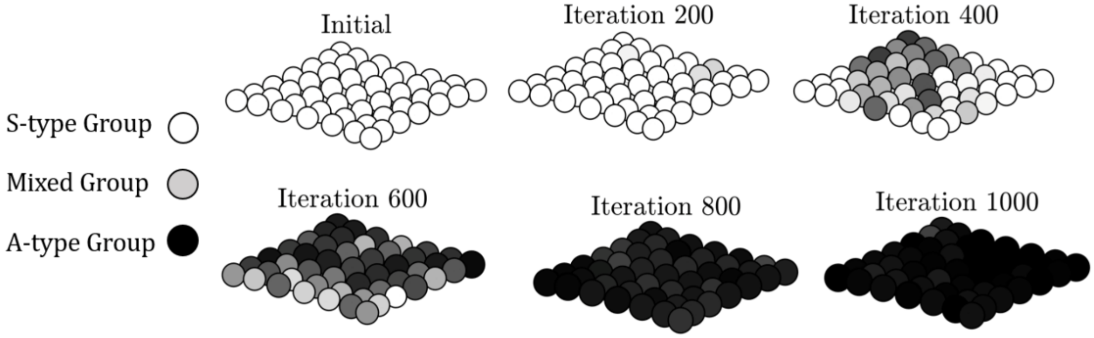

Fig. S1 Propagation of the A-type under selective migration when $\delta = 0.9998$ and $R_{in} = 500$. Each panel depicts the 7×7 metapopulation grid. Bubbles indicate the spatial location of groups. The proportion of A-types in a group is represented by hue, with $\pi = 0$ (an S-type group) indicated in white and $\pi = 1$ (an A-type group) in black. Bubble diameter is proportional to the logarithm of group size. Starting from an S-type metapopulation the A-type can arise by mutation and drift and propagate across the metapopulation by random migration as depicted because the cost of altruism is so slight as to make the selection regime nearly neutral.
